# Circulating Fibroblast Growth Factor-2, HIV-Tat, and Vascular Endothelial Cell Growth Factor-A in HIV-Infected Children with Renal Disease Activate Rho-A and Src in Cultured Renal Endothelial Cells

**DOI:** 10.1371/journal.pone.0153837

**Published:** 2016-04-20

**Authors:** Jharna R Das, J. Silvio Gutkind, Patricio E Ray

**Affiliations:** 1 Center for Genetic Medicine Research, Children’s National Health Systems, Washington DC, United States of America; 2 Department of Pharmacology, Moores Cancer Center, University of California San Diego, La Jolla, California, United States of America; 3 Division of Nephrology, Children’s National Health Systems, Washington DC, United States of America; 4 Department of Pediatrics, The George Washington University, Washington DC, United States of America; Candiolo Cancer Institute, ITALY

## Abstract

Renal endothelial cells (REc) are the first target of HIV-1 in the kidney. The integrity of REc is maintained at least partially by heparin binding growth factors that bind to heparan sulfate proteoglycans located on their cell surface. However, previous studies showed that the accumulation of two heparin-binding growth factors, Vascular Endothelial Cell Growth Factor-A (VEGF-A) and Fibroblast Growth Factor-2 (FGF-2), in combination with the viral protein Tat, can precipitate the progression of HIV-renal diseases. Nonetheless, very little is known about how these factors affect the behavior of REc in HIV+ children. We carried out this study to determine how VEGF-A, FGF-2, and HIV-Tat, modulate the cytoskeletal structure and permeability of cultured REc, identify key signaling pathways involved in this process, and develop a functional REc assay to detect HIV+ children affected by these changes. We found that VEGF-A and FGF-2, acting in synergy with HIV-Tat and heparin, affected the cytoskeletal structure and permeability of REc through changes in Rho-A, Src, and Rac-1 activity. Furthermore, urine samples from HIV+ children with renal diseases, showed high levels of VEGF-A and FGF-2, and induced similar changes in cultured REc and podocytes. These findings suggest that FGF-2, VEGF-A, and HIV-Tat, may affect the glomerular filtration barrier in HIV+ children through the induction of synergistic changes in Rho-A and Src activity. Further studies are needed to define the clinical value of the REc assay described in this study to identify HIV+ children exposed to circulating factors that may induce glomerular injury through similar mechanisms.

## Introduction

HIV-infected children are at risk of developing several renal diseases, including HIV-associated nephropathy (HIVAN), Hemolytic Uremic Syndrome, Thrombotic Thrombocytopenic Purpura, and acute kidney injury [1]. Although these renal diseases are triggered by different etiological factors, they cannot be prevented and treated successfully without decreasing the viral load [2]. Previous studies showed that HIV-1 affects the survival, growth, and differentiation of renal epithelial cells [3, 4]. However, the tubulo-reticular inclusions detected in patients with HIVAN suggest that renal endothelial cells (REc) are also an important target of HIV-1 [5]. Moreover, despite the fact that changes in the cytoskeletal structure of REc may facilitate the collapse of glomerular capillaries, very little is known about how cytokines and viral proteins released by HIV-infected cells can affect the outcome of these lesions.

Heparan sulfate proteoglycans (HSGP) expressed on the surface of glomerular endothelial cells act as low affinity receptors for heparin binding growth factors, and play a key role maintaining the cytoskeletal and integrity of these cells [6, 7]. During inflammatory diseases, HSPG increase the binding and recruitment of cytokines and mononuclear cells [8], and these events facilitate the accumulation of viral proteins and heparin binding growth factors in renal glomeruli. Subsequently, these heparin-binding growth factors are accumulated the kidney and excreted in the urine [9]. In support of this notion, previous studies in HIV+ children and HIV-Tg_26_ mice with renal diseases showed an up-regulated expression of renal HSPG [10, 11], and high urinary levels of Vascular Endothelial Growth Factor-A (VEGF-A) and Fibroblast Growth Factor-2 (FGF-2) were detected in patients with HIV-renal diseases [9, 11–14]. In addition, the HIV-1 transactivator of transcription (Tat) protein, which is released by HIV-infected cells and taken up by endothelial cells, also functions as a heparin binding growth factor [15, 16]. In this manner, extracellular Tat can act in synergy with VEGF-A or FGF-2 to modulate the cytoskeletal structure of endothelial cells [17] and podocytes [18, 19]. Furthermore, HIV-1 binds to HSPG through electrostatic interactions that involve the positively charged domains of gp120 and the negative charges of HSPG on endothelial cells [20], and these interactions increase virus infectivity and facilitate the release of HIV-Tat [21]. In summary, these findings provide compelling evidence to suggest that VEGF-A, FGF-2, and HIV-Tat, acting in synergy, may play important roles modulating the cytoskeletal structure and permeability of RGEc in HIV+ children.

Previous studies suggest that the Rho family of GTPases [22] play an important role modulating the cytoskeletal structure and permeability of endothelial cells. GTPases are molecular switches that cycle between active (GTP-bound) or inactive (GDP-bound) states [22–26] and regulate several endothelial cell behaviors, including angiogenesis, cell adhesion, migration, and permeability. Thus, a more complete knowledge of the pathogenesis of HIV-renal diseases cannot be obtained without understanding how FGF-2, VEGF-A, and HIV-Tat modulate the Rho family of GTPases in REc. Therefore, we carried out this study to determine how these factors affect the cytoskeletal structure and permeability of cultured human REc, identify key signaling pathways involved in this process, and develop a functional REc assay to identify HIV+ children exposed to circulating factors that induce similar cytoskeletal and permeability changes.

## Material and Methods

### Reagents

The reagents described below were obtained from the following sources: human recombinant VEGF-165 (PeproTech (Rocky Hill, NJ); HIV-1 Tat protein (NIH AIDS Reagent Program); human recombinant FGF-2 (R&D Systems); Heparin derived from porcine intestinal mucosa, USP 5,000 USP (Units /ml) APP Pharmaceuticals LLC; SU6656 (Calbiochem); C3 Transferase (List Biological Labs, Campbell, CA); Y-27632, cyclic 3’5 monophosphate (cAMP) analog, thrombin, DAPI and Beta-actin mouse monoclonal antibody (Sigma-Aldrich, MO); RO 20–1724 (MyBiosource San Diego), and RhoA (67B9) rabbit monoclonal antibody, phospo-p44/42 MAP kinase (Thr202/Tyr204), p44/42 MAP kinase, Src rabbit monoclonal antibody, phospho-Myosin Light Chain 2 (Thr/Ser 19) rabbit polyclonal antibody, Myosin Light Chain 2 rabbit polyclonal antibody, and VEGF receptor 2 (VEGFR2) 55B11 rabbit monoclonal antibody, were all obtained from Cell Signaling Technology (Danvers, MA). The anti-Rac1 mouse monoclonal antibody was from BD Transduction Laboratories^™^, and the phospho-Src [pY418] or (pp60src) rabbit polyclonal antibodies were from (Invitrogen). The VE-cadherin CD144 clone BV6 mouse monoclonal antibody was from EMD Millipore, MA); human Von Willebrand (vWF) antibody from DAKO (Carpinteria, CA), CD31 for R&D System for immunofluorescence, and Abcam, (Cambridge, MA) for Western blots. The goat anti-rabbit IgG-HRP and the goat anti-mouse IgG-HRP, were from (Bio-Rad, CA). Rhodamine goat anti-mouse, Rhodamine goat anti-rabbit, Alexa Fluor 488-labeled phalloidin, and FITC-dextran were all obtained from (Molecular Probes, Inc.)

### Generation and characterization of the human glomerular endothelial cells line (HGEc-1)

**Fig 1:**
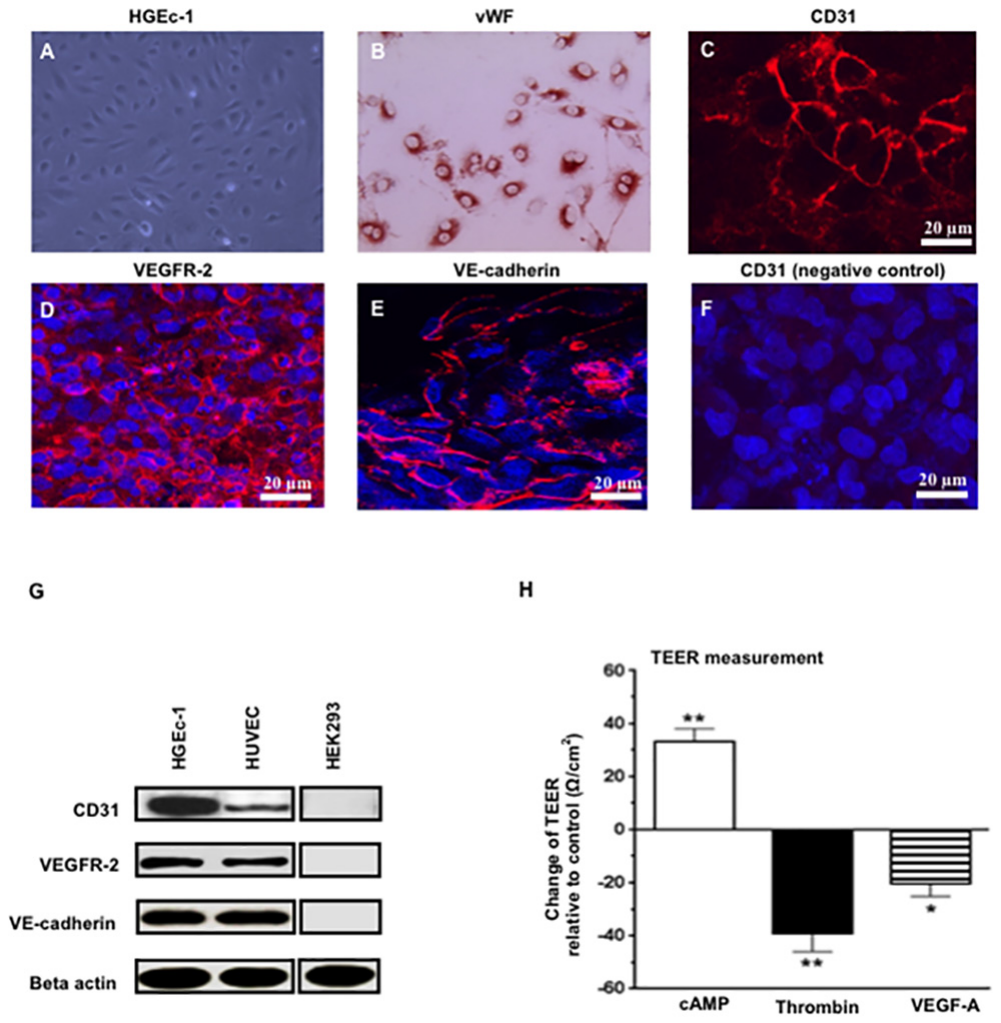
Characterization of the human glomerular endothelial cell line (HGEc-1). **(A)** Phase contrast microscopy picture of cultured HGEc-1, Magnification, X100. **(B)** Immunohistochemistry staining of cultured HGEc-1 expressing the endothelial cell marker Von Willebrand Factor (vWf), red color, Magnification, X100. Immunofluorescence staining of cultured HGEc-1 for **(C)** CD31, **(D)** VEGFR-2, **(E)** VE-cadherin, all in red color, and **(F)** human podocytes incubated with the anti- CD31 antibody (negative controls). Cell nuclei are visualized with DAPI in blue color. Scale bar = 20 μm. **(G)** Western blots show CD31, VEGFR2, VE-cadherin, and Beta actin expression in cultured HGEc-1, human umbilical vein endothelial cells (HUVEC), and human renal embryonic epithelial cells (HEK293). **(H)** Changes in trans-endothelial electrical resistance (TEER) induced by the cyclic AMP analogue 8-pCPT-cAMP (30 μm) in combination with the cAMP- phosphodiesterase inhibitor RO-20-1724 (20 μm); thrombin (100 units/ml), or VEGF-A (50 ng/ml). Results are expressed as changes relative to controls. Bar graphs show mean ± SEM corresponding to three different experiments. Values significantly different from control were marked with *asterisk*, *p<0.05 and **p<0.01.

Primary HGEc isolated by Cell Systems (ACBRI, Seattle, Washington USA), were characterized, and cultured as previously described [27]. To generate the HGEc-1 cell line, these cells were transfected with the pSVT vector containing the coding sequences of the SV40 large T (kindly provided by Dr. A Srinivasan, from the Wistar Institute, Philadelphia, PA), as described before [28]. Subsequently, several transformed clones were transduced with 2x10^9^ particles/ml of adenoviral vectors (rAd)-carrying the catalytic subunit of human telomerase (hTERT) (from Applied Biological Materials, Inc. Richmond, BC, Canada) and cultured for 6–8 additional weeks. These cells express vWF, VE-cadherin, CD31, and VEGFR2, and formed monolayers whose trans-endothelial electrical resistance (TEER) was ~ 60 ± 10 Ω /cm^2^ after one week in culture. In addition, these cells responded to cyclic 3’5 monophosphate (cAMP) analogs, thrombin, and VEGF-A (Fig 1H) in a similar manner as primary REc [29]. HGEc-1 were cultured in DMEM media with 10% FBS without endothelial cell growth factor supplements. The podocyte cell line used in this study (P-2) was generated from a child with HIVAN as described in detail before [30]. Briefly, these cells were immortalized with adenoviral vectors carrying DNA sequences encoding the SV-40 large T antigen and human telomerase. P-2 cells express the podocyte specific markers WT-1, synaptopodin, nestin, and nephrin, and show similar changes in Rho-A activity when compared to primary podocytes exposed to FGF-2 and HIV-Tat [30]. Podocytes were cultured in DMEM supplemented with 10% fetal bovine serum (FBS), 100 units /ml of penicillin, 100 mg/ml of streptozocin, and 0.25 mg/ml of amphotericine B.

### Trans-endothelial electrical resistance (TEER) and renal endothelial permeability assay

TEER was measured using a EVOM voltohmeter connected to an Endohm 9 electrode chamber (World Precision Instruments, Sarasota, FL) as previously described [29]. Tissue culture inserts containing monolayers of HGEc cultured on Transwell-Collagen-coated membrane, were placed in the chamber and the TEER was recorded after 10 sec [29]. The permeability experiments were done as described before [31]. Briefly, 3 x 10^5^ HGEC-1 were plated onto Transwell Collagen-coated membrane inserts (Corning Costar, Cat No 3415) in DMEM media with 10% FBS, and left for 3 days to form mature monolayer with a trans-endothelial electrical resistance (TEER) of ~ 60 ± 10 Ω /cm^2^. Subsequently, the cells were starved for 5 hours in serum free DMEM media without phenol red, treated with the corresponding reagents, and incubated with 1 mg/ml FITC-dextran (Molecular Probes, D-1844, Invitrogen) for 30 min at 37°C. Samples collected from the bottom chambers were read in triplicates on the Victor 3V1420 multi-counter (Perkin-Elmer, Wellesley, PA).

### GST Pull-Down assays, Western blots, and transfections with Rho-A DNA construct

Rho-A and Rac1 activation was assessed by GST pull-downs using GST-Rhotekin and PAK-CRIB recombinant protein respectively bound to glutathione slurry resin as we have previously described [30–32]. Western blots were performed using standard techniques as described before [30]. Equal amount of protein (cell lysate without bound GST beads) were used to estimate total Rho-A/Rac1. Results were detected using Supersignal West Pico Chemiluminescent Substrate, from (Thermo Scientific, IL). The images were captured on Kodak film (X-OMAT) from Kodak Scientific Imaging. All experiments were repeated three times, and quantified by densitometric analysis using Adobe Photoshop 6.0. To assess the specific role of Rho-A, HGEc-1 cells were transfected using Lipofectamine 2000 (Invitrogen), with the pCEFL-MCherry control plasmid, the pCEFL-AU5-RhoAQL plasmid carrying a constitutively active Rho, or the pCEFL-AU5-RhoAN19 plasmid carrying a dominant negative mutant Rho-A. These constructs were generated and validated as described in previous studies [31, 33, 34].

### Immunostaining

Immunofluorescence studies were done as described before [31]. Changes in F-actin were assessed using phalloidin. Cell nuclei were visualized with Hoechst 33342 (Invitrogen), and observed under an Axioplan 2 (Zeiss) confocal laser-scanning microscope. Stress fibers were counted using ImageJ software.

### Collection of Urine

These experiments were approved by the Institutional Review Board (IRB) of Children’s National Medical Center (protocol # 00000002), and carried out in accordance with the principles of the Declaration of Helsinki. Urine samples were collected from HIV-infected children with and without renal diseases (n = 5 per group), after obtaining written or verbal consent from the patients or their parents, legal guardians, or caretakers on behalf of the younger children. An Information Sheet explaining the purpose of the study was provided in each case as requested by Children’s IRB that approved the consent procedure. Urine samples were given a code number, and the records were kept anonymous to prevent the identification of the patients. Urine samples were centrifuged to clear cell debris, and the supernatants were kept frozen at -70°C until used. Samples were screened for endotoxin and those containing > 0.1 ng/μg protein were excluded. For the permeability assays the urine samples were diluted, and control and HIV+ samples containing similar values of urinary creatinine (~ 10 mg/dl) were compared. The urinary levels of FGF-2 and VEGF-A were measured using quantitative sandwich enzyme-linked immunosorbent assay (ELISA) kits for FGF-2 and VEGF-A (Quantikine, R & D System, Minneapolis MN, USA), as previously described [9, 35], and the results were expressed as a ratio of the urinary creatinine values.

### Statistical Analysis

All data were from at least three independent experiments and statistical analysis was performed using Prism 6. For parametrically distributed data, we used the Student’s or ANOVA when more than two groups were compared. When the data were not normally distributed we used the non-parametric Mann Whitney *U* test, or the Kruskal-Wallis test when more that two groups were compared. P values < 0.05 were considered statistically significant.

## Results

### Generation of a human glomerular endothelial cell line (HGEc-1) and permeability assay

Since primary HGEc have a limited life span and require serum and angiogenic growth factors to survive in culture, we generated a HGEc line to assess the behavior of REc in the absence of serum and angiogenic growth factors. Briefly, as described in the methods section, primary HGEc were transfected with DNA carrying the simian virus 40-(SV40) T antigen and infected with adenoviral vectors carrying the Telomerase reverse transcriptase protein (TERT). Colonies of immortalized REc (HGEc-1) showing typical endothelial morphology were selected and expanded. HGEc-1 express VEGFR-2, and stained positive for the endothelial cell markers vWF, VE-cadherin, and CD31 (Fig 1). Furthermore, they form tight monolayers in tissue culture, and show changes in permeability and trans-endothelial electrical resistance (TEER) (Fig 1). These changes are similar to those seen in primary HGEc treated with cAMP analogues, thrombin, and VEGF-A [29].

### VEGF-A or FGF-2, in combination with HIV-Tat increases the permeability of HGEc-1 through the activation of Rho-A, phospho-myosin light chain (pMLC), and Src

**Fig 2:**
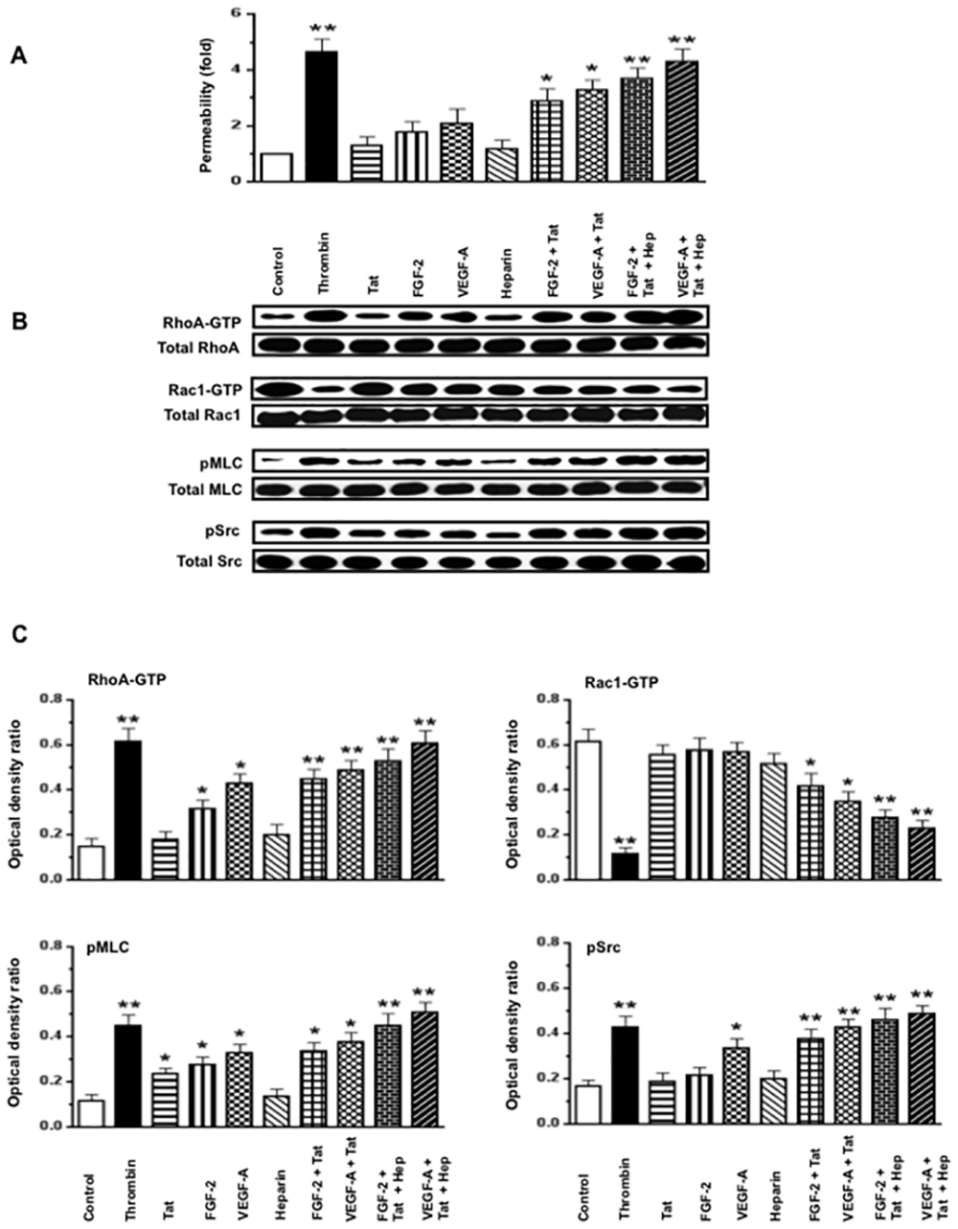
FGF-2 and VEGF-A in combination with HIV-Tat and heparin increase the permeability of cultured HGEc-1 through Rho-A and Src dependent pathways. **(A)** Monolayers of 5 hours-starved HGEc-1 were stimulated by thrombin (100 units/ml) as a positive control, Tat (100 ng/ml), FGF-2 (50 ng/ml), VEGF-A (50 ng/ml), and Heparin (50 units/ml) alone or in combination. The data represent FITC-dextran permeability changes expressed as fold increase. **(B)** Overnight-starved HGEc-1 monolayer were treated for 5 min as described above and then harvested to assess the phosphorylation of Rho-A, Rac1, MLC, and Src, as described in Methods. Panel B shows representative Western blots corresponding to the phosphorylation changes. **(C)** The graphs show mean ± SEM values corresponding to three different Western blots that assessed the phosphorylation of Rho-A, Rac1, MLC, and Src in cultured HGEc-1. Results were expressed in arbitrary optical density units as a ratio of the total activity. Values significantly different from control cells treated with serum free media were marked with *asterisk*, *p<0.05 and **p<0.01.

**Fig 3:**
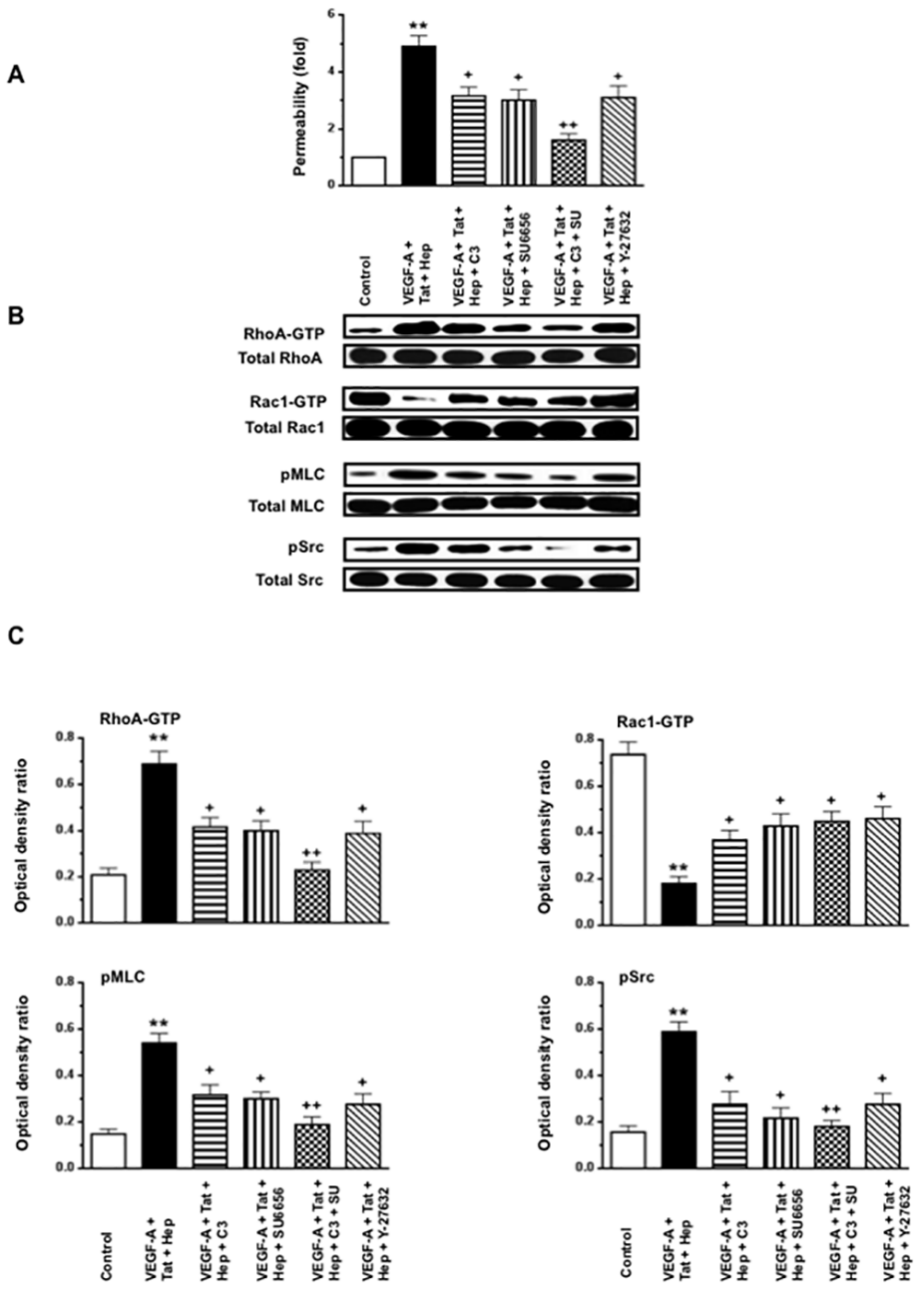
VEGF-A in combination with HIV-Tat and heparin, increases the permeability of cultured HGEc-1 through Rho-A and Src dependent pathways. **(A)** Monolayers of 5 hours-starved HGEc-1 were stimulated by VEGF-A (50 ng/ml), Tat (100 ng/ml), and Heparin (50 units/ml) all combined. The Rho-A inhibitor: C3 transferase (20 ng/ml), was added 4 hours before stimulation and the inhibitor of Src family kinase SU6656 (1 μM) and ROCK inhibitor Y-27632 (10 μM) were added 1hr before stimulation. The data represent FITC-dextran permeability expresses as fold increase. **(B)** Overnight-starved HGEc-1 monolayers were treated for 5 min as described above and then harvested to assess the phosphorylation of Rho-A, Rac1, MLC and Src, as described in Methods. Panel B shows representative Western blots corresponding to the phosphorylation changes. **(C)** The graphs show mean ± SEM values corresponding to three different Western blots that assessed the phosphorylation of Rho-A, Rac1, MLC, and Src in cultured HGEc-1. Results were expressed in arbitrary optical density units expressed as a ratio of the total activity. Values significantly different from control cells treated with serum free media were marked with *asterisks* **p<0.01, and those significantly different from the cells treated with VEGF-A + Tat + Heparin were marked with crosses, + p<0.05 and ++ p<0.01.

**Fig 4:**
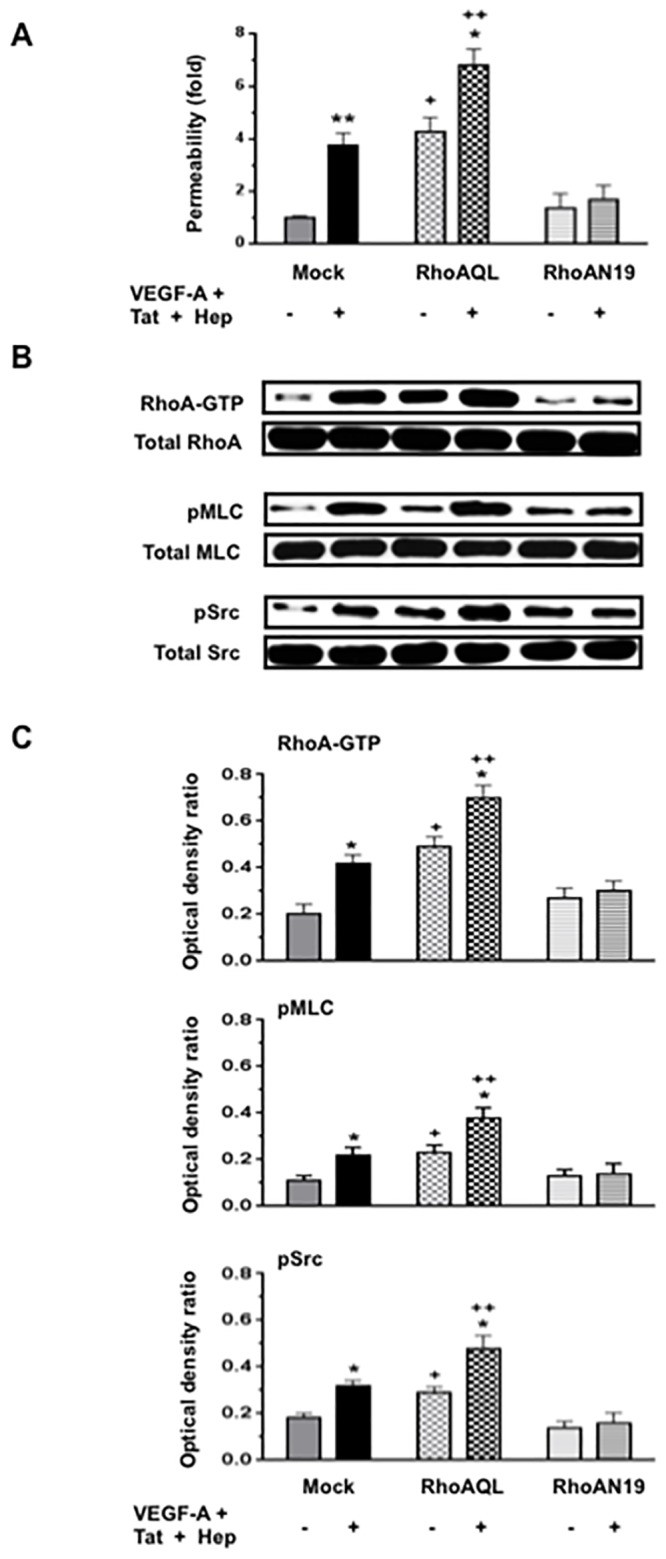
Rho-A activation increases the permeability of HGEc-1. **(A)** Cultured HGEc-1 cells were transfected with different plasmids, pCEFL-mock, constitutively active RhoAQL (pCEFL-AU5-RhoAQL), or dominant negative mutant RhoAN19 (pCEFL-AU5-RhoAN19). Twenty-four hours later, the cells were treated with VEGF-A (50 ng/ml) + Tat (100 ng/ml) + Heparin (50 units/ml), all together, and exposed to FITC-dextran as described in methods. **(B)** In other experiments HGEc-1 cells were treated for 5 min as described above and then harvested to assess the phosphorylation of Rho-A, MLC, Src as described in Methods. Panel B shows representative western blots corresponding to the phosphorylation changes. **(C)** The graphs show mean ± SEM values corresponding to three different Western blots that assessed the phosphorylation of Rho-A, MLC and Src in cultured HGEc-1. Results were expressed in arbitrary optical density units as a ratio of the total activity. In each group, mock, RhoAQL and RhoAN19 transfected cells were treated with either serum free media (Controls) (-), or VEGF-A + Tat + Heparin (+). Groups that were significantly different from controls (-) were labeled with *asterisk*, *p<0.05 and **p<0.01. Cells transfected with constitutively active RhoAQL that were significantly different from mock or RhoAN19 cells, were labeled with crosses: + p <0.05 and ++ p<0.01.

Initially, we explored how VEGF-A, FGF-2, HIV-Tat, and heparin, alone or in combination, affected the permeability of cultured HGEc-1 (Fig 2). Thrombin was used as a positive control [31], because it induces permeability changes through Rho-A activation. Heparin was used to mimic the effects of endogenous HSPGs, which prevent the degradation of heparin binding growth factors, and could either enhance or inhibit the angiogenic activity of FGF-2 or VEGF-A [36–41]. As described before in primary HGEc [28], we found that VEGF-A induced modest permeability changes in HGEc-1 (Fig 2). HIV-Tat or heparin, acting alone, did not induce permeability changes in cultured HGEc-1. However, VEGF-A or FGF-2, in combination with HIV-Tat, induced significant permeability changes that were further increased by heparin (Fig 2). Subsequently, considering that VEGF-A or FGF-2 in combination with HIV-Tat and heparin induced the most significant permeability changes acting through similar signaling pathways, all follow up experiments were done in cells treated with VEGF-A+ HIV-Tat + heparin. As shown in Fig 3, we confirmed that the permeability changes were mediated through activation of the Rho-A, pMLC, and c-Src pathways, and prevented, at least partially, by C3-Transferase, SU6656, or Y-27632, which inhibited the activity of Rho-A, Scr, and ROCK respectively. In agreement with previous studies done in other endothelial cell types, we confirmed that the activation of Rho-A and Rac-1 changed in opposite directions. Moreover, we found that an active Rho-A construct was sufficient to increase the permeability of HGEc-1, although this construct also increased Src activity (Fig 4). Taken together, our findings support the notion that cross-talk between Rho-A and Src play a critical role modulating the permeability activity of VEGF-A + HIV-Tat + heparin in cultured HGEc [24].

### FGF-2 and VEGF- A, in combination with HIV-Tat and heparin, induce the formation of stress fibers through activation of Rho-A in HGEc-1

**Fig 5:**
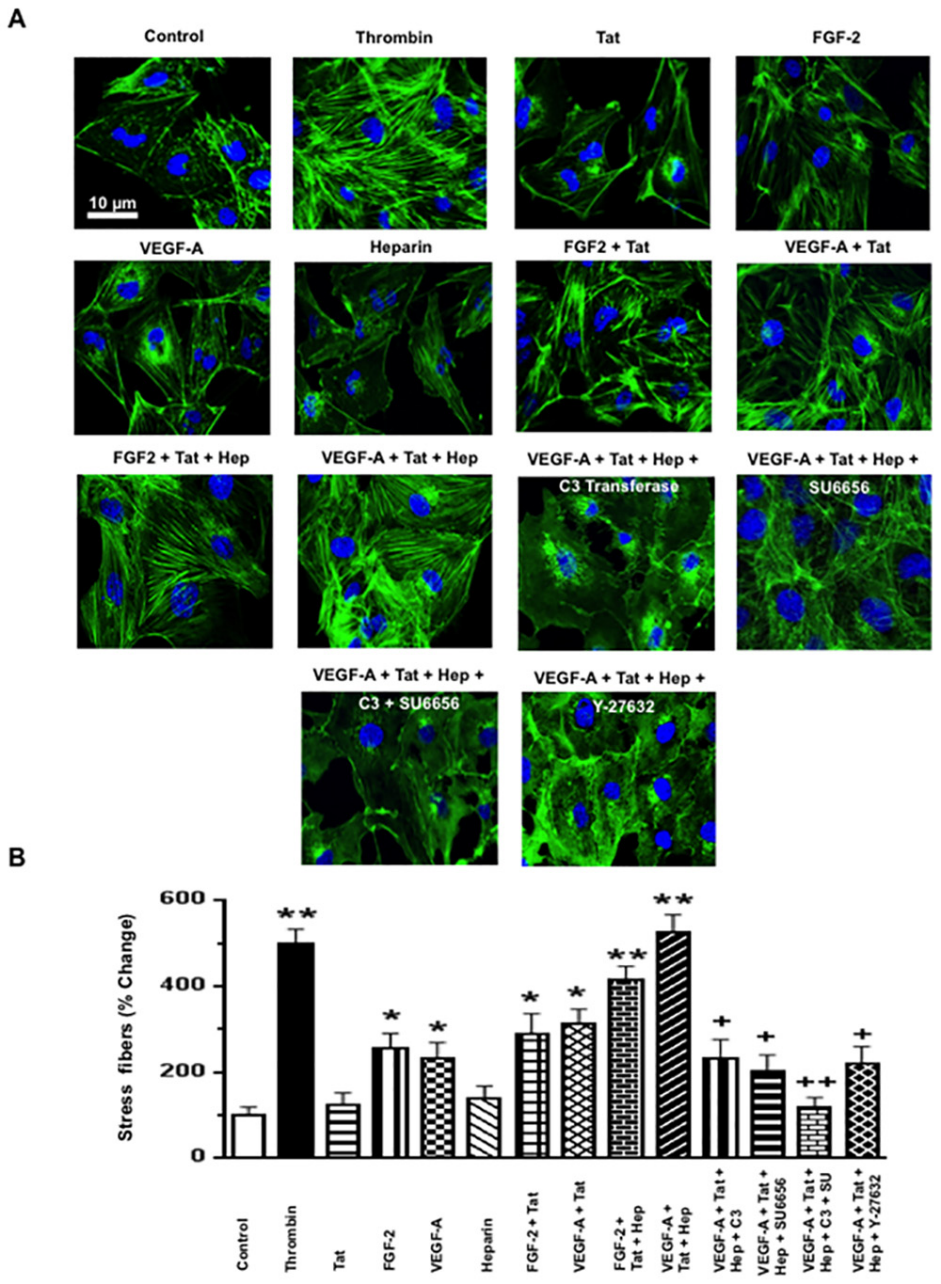
FGF-2 and VEGF-A, in combination with HIV-Tat and heparin, induce the formation of stress fibers through Rho-A dependent pathways. **(A)** Panel A shows representative changes in the formation of stress fibers detected in cultured HGEc-1. Overnight-starved HGEc-1 monolayer were stimulated by thrombin (100 units/ml) as a positive control, Tat (100 ng/ml), FGF-2 (50 ng/ml), VEGF-A (50 ng/ml), and Heparin (50 units/ml) alone or in combination. The RhoA inhibitor: C3 transferase (20 ng/ml), was added 4 hrs. before stimulation and the inhibitor of Src family kinase SU6656 (1 μM) and ROCK inhibitor Y-27632 (10 μM) were added 1hr before stimulation and 20 min after treatment, F-actin fibers were visualized in cells by staining with 2 μg/ml of Alexa Fluor 488-labeled phalloidin. Cell nuclei were stained with Hoechst 33342. The scale bar is 10 μm. **(B)** The graphs show mean ± SEM values corresponding to the formation of stress fibers in three different experiments. Results were expressed as % changes in stress fibers formation relative to control cells. Values significantly different from controls were marked with an *asterisk*, *p<0.05 and **p<0.01, and those different from cells treated with VEGF-A + Tat + Heparin were marked with a cross, +p <0.05 and ++p<0.01.

**Fig 6:**
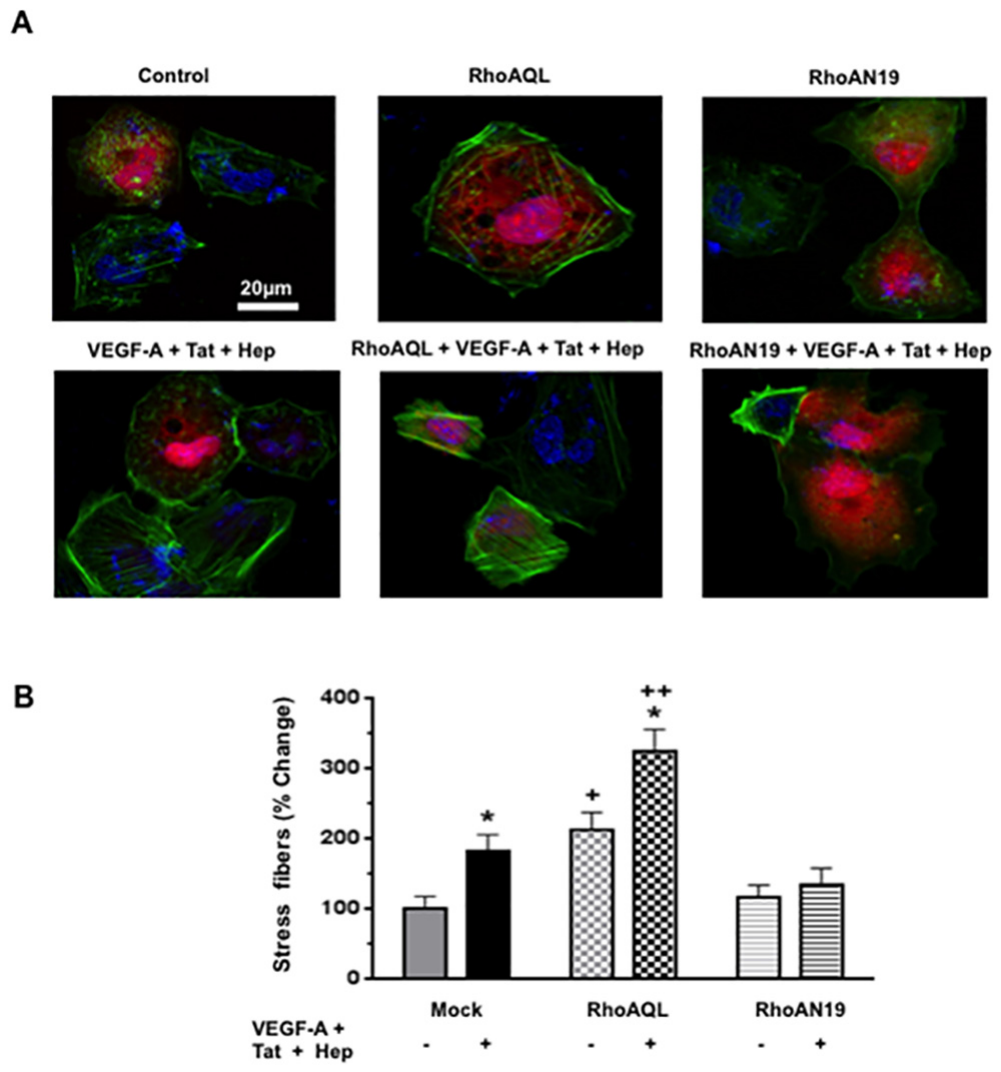
Rho-A activation increases the formation of stress fibers in HGEc-1. **(A)** HGEc-1 were transfected with the corresponding Rho-A constructs described above. Subsequently, 24 hours later, they were seeded on coverslips, treated with (VEGF-A + Tat + Heparin), and stained with F-actin to visualize the formation of stress fibers as described above. Scale bar = 20 μm. **(B)** The graphs show mean ± SEM values corresponding to three different experiments that assessed the formation of stress fibers in cultured HGEc-1. Results were expressed as % changes in stress fibers relative to controls. Control cells (-) were treated with serum free medium. Statistically significant differences between control cells (-) vs. VEGF-A + Tat + Heparin-treated cells (+), were highlighted with an *asterisk*, *p<0.05. Difference between cells treated with VEGF-A + Tat + Heparin vs. other groups were highlighted with a cross, ++ p < 0.01.

Then, we explored whether the permeability and signaling changes described above were associated with changes in the cytoskeletal structure of HGEc-1. We found that HGEc-1 treated with thrombin alone, FGF-2 + Tat, VEGF-A + Tat, or both factors in combination with heparin, increased the number of central stress fibers running along the longitudinal axis of HGEc1 (Fig 5). These changes were inhibited with either C3-transferase from *Clostridium botulinum*, which blocks Rho-A activity, SU6656, which blocks Src activity, and the ROCK inhibitor Y27632 (Fig 5). Taken together, our findings suggest that Rho-A, MLC, and Src activation play a critical role inducing the formation of central stress fibers in cultured HGEc-1. To confirm that Rho-A activation per se affected the formation of central stress fibers, cultured HGEc-1 were transfected with DNA constructs encoding the MCherry red fluorescent protein (pCEFL-MCherry), in combination with plasmids encoding a constitutively active Rho-A (pCEFL-AU5-RhoAQL) or a dominant negative mutant Rho-A (pCEFL-AU5-RhoAN19) (Fig 6), as described in previous studies [31, 33, 34]. These experiments showed that Rho-A activation per se induced the formation of stress fibers, both in the presence and absence of VEGF-A + Tat + heparin (Fig 6).

### Urine samples harvested from HIV-infected children with renal diseases increase the permeability of cultured HGEc-1 and podocytes through the activation of Rho-A and Src

**Fig 7:**
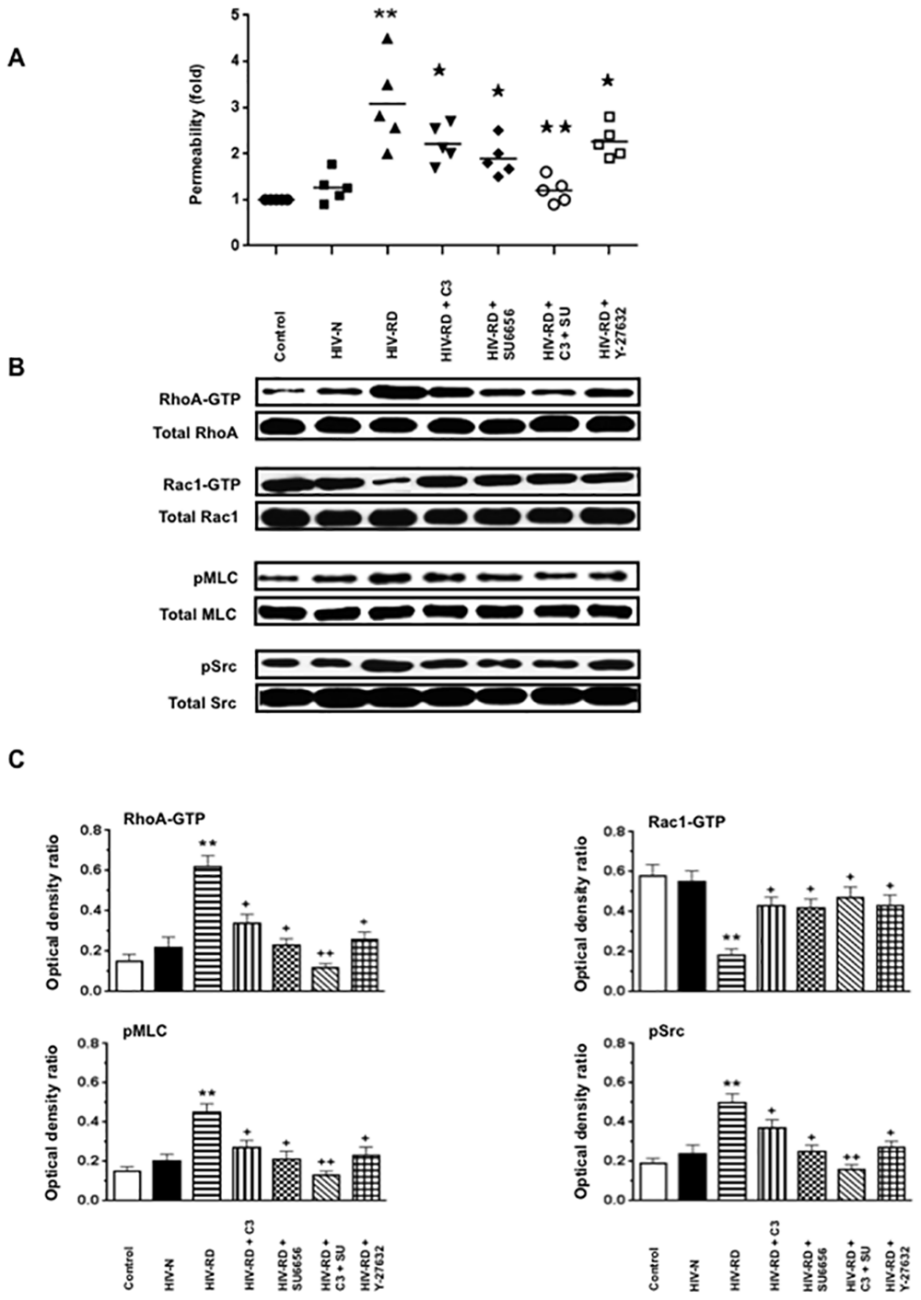
Urine samples harvested from HIV-infected children with renal diseases increase the permeability of cultured HGEc-1 through RhoA and Src mediated mechanisms. **(A)** Urine samples harvested from HIV infected children with and without renal diseases (RD) were used (1:10 dilution) to stimulate monolayers of 5 hours-starved HGEc-1. The RhoA inhibitor: C3 transferase (20 ng/ml), was added 4 hrs before stimulation. The inhibitor of Src family kinase SU6656 (1 μM) and the ROCK inhibitor Y-27632 (10 μM) were added 1hr before stimulation. The data represent FITC-dextran permeability expresses as fold increase. **(B)** Overnight-starved HGEc-1 monolayer were treated with urine (1:20 dilution) for 5 min as described above and then harvested to assess the phosphorylation of RhoA, Rac1, MLC, and Src, as described in Methods. **(C)** The graphs show mean ± SEM values corresponding to three different Western blots that assessed the phosphorylation of Rho-A, Rac1, MLC, and Src, in cultured HGEc-1. Results were expressed in optical density units expressed as a ratio of the total activity. Values significantly different from the control group (HIV-N; n = 5) were marked with *asterisks* **p<0.01, and those significantly different from the HIV-RD group (n = 5) were marked with stars ★ p<0.05 and ★★ p<0.01 (for permeability), or crosses, + p <0.05 and ++ p<0.01 (for signaling).

**Fig 8:**
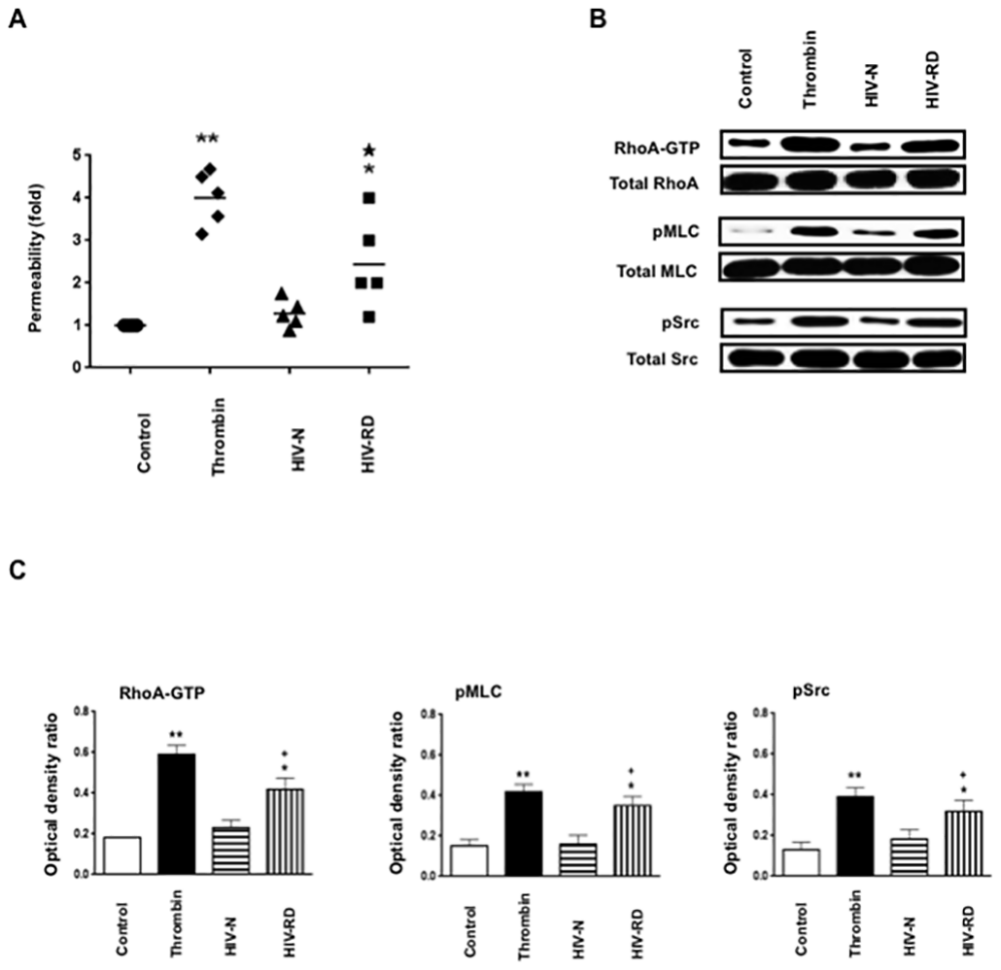
Urine samples harvested from HIV-infected children with renal diseases increase the permeability of cultured podocytes cell line (P-2) through Rho-A and Src mediated mechanisms. **(A)** Urine samples harvested from HIV infected children with and without renal diseases (RD) were used (1:10 dilution) to stimulate monolayers of 5 hours-starved P-2 cells. The data represent FITC-dextran permeability changes expressed as fold increase. **(B)** Overnight-starved P-2 monolayers were treated with urine (1:20 dilution) for 5 min as described above and then harvested to assess the phosphorylation of Rho-A, MLC, and Src, as described in Methods. **(C)** The graphs show mean ± SEM values corresponding to three different Western blots that assessed the phosphorylation of Rho-A, MLC, and Src in cultured podocytes. Results were expressed in optical density units expressed as a ratio of the total activity. Values significantly different from the serum free treated control cells (Control) were marked with *asterisk*, *p<0.05 and **p<0.01, and differences between HIV-RD (n = 5) and HIV-N (n = 5) urine samples, were marked with stars ★ p< 0.05 (for permeability) or crosses + p<0.05 (for signaling).

Previous studies done in patients with HIV-renal diseases reported high plasma and urine levels of FGF-2 and VEGF-A [9, 11, 14]. Therefore we collected urine samples from HIV+ children with (HIV-RD) or without renal diseases (HIV-N), and measured the levels of FGF-2 and VEGF-A. As expected, the urinary levels of FGF-2 and VEGF-A were elevated in children with HIV-RD when compared to HIV-N (FGF-2 = 27.34 ± 17.61 vs. 3.44 ± 2,15 mean ± SD pg/μg urinary creatinine (UCr); p < 0.01; VEGF-A = 1613 ± 1590 vs. 38.40 ± 21.45 mean ± SD pg/μg UCr; p < 0.0079; for HIV-RD vs. HIV-N respectively, n = 5 per group). In addition, we found that HIV-RD samples increased the permeability of cultured renal endothelial cells and podocytes through Rho-A and Src mediated pathways that were inhibited by Rho-A, Src, and ROCK inhibitors (Figs 7 and 8). These changes were not affected by the low levels of endotoxin detected in the urine samples (< 25 pg/ml; S1 Fig). Taken together, these findings suggest that FGF-2 and VEGF-A can affect the cytoskeletal structure and permeability of REc and podocytes acting through Rho-A and Src dependent mechanisms.

## Discussion

Renal endothelial cells are a target of circulating viral proteins and heparin binding cytokines released by HIV-infected cells. Therefore, it is important to understand how these factors affect the cytoskeletal structure and permeability of REc. In the current study we found that FGF-2 and VEGF-A, in combination with HIV-Tat and heparin, induced cytoskeletal changes and increased the permeability of cultured HGEc acting through synchronized changes in Rho-A, Src, and Rac-1 activity. Furthermore, we found that urine samples collected from HIV+ children with renal diseases induce similar changes in cultured HGEc and podocytes. Overall, these findings suggest that FGF-2, VEGF-A, and HIV-Tat, released by HIV-infected cells, may affect the integrity of the glomerular filtration barrier acting through synchronized changes in Rho-A, Rac-1, and Src activity.

In this study, we explored the role of FGF-2 and VEGF-A, because both heparin-binding growth factors are accumulated in the kidney of children with HIV-renal diseases [9, 11–14], and affect the outcome of HIV-nephropathy in HIV-Tg mice [10, 12, 30] and rats [42, 43]. As discussed before, HSPG act as low affinity receptors increasing the accumulation of HIV-1 Tat [44], as well as the binding of VEGF-A and FGF-2 to their high affinity tyrosine kinase receptors [6, 7]. Although FGF-2 and VEGF-A act through different high affinity receptors, there is substantial crosstalk between FGF receptors (FGFR) and vascular endothelial cell growth factor receptors (VEGFR) [45–49]. VEGF-A and HIV-Tat induce permeability changes acting through the KDR/Flk-1 (VEGR2) [15]. In addition, they regulate cell adhesion and angiogenic behaviors acting through α_v_β_5_ integrins [49], which interact with the Rho family of GTPases [50]. In contrast, FGF-2 does not induce permeability changes acting alone, but increases the permeability and angiogenic activity of HIV-Tat, probably acting through the stimulation of FGFR and VEGFR2 receptors that induce changes in Rho-A and Src activity, and the expression of α_v_β_3_ integrins [49]. Previous studies showed that VEGF-A decreases the TEER in primary HGEc, facilitating the transit of water and small molecules, but did not increase the permeability to macromolecules [29]. We confirmed these findings, but also found that in the presence of HIV-Tat or heparin, VEGF-A increases the permeability of HGEc to large macromolecules. Heparin mimics the action of HSPG, facilitating the binding of VEGF-A to the VEGFR-2 [7], and blocking the cellular uptake of Tat [44], therefore, prolonging its interactions with VEGFR2 on the cell surface [15, 16]. In agreement with this notion, the most significant permeability changes were found when VEGF-A, HIV-Tat, and heparin were all used together.

The integrity of endothelial cell monolayers is maintained through the regulation of the size of intercellular gaps. This process is controlled in tightly regulated manner through changes in Rho-A and Rac-1 activity [22, 51–53]. Rho-A activation induces the contraction of actin and myosin stress fibers, increasing the centripetal tension, the size of intercellular gaps, and increasing the permeability of endothelial cells [22, 51–53]. In contrast, Rac-1 resists this tension, decreasing the permeability of REc by increasing the preservation of the VE-cadherin adherens junctions between neighboring endothelial cells [51–53]. In agreement with this notion, we found that Rho-A and Rac-1 changed in opposite directions in correlation with the permeability changes. The cytoskeletal distribution of actin and myosin fibers in cultured endothelial cells reflect this balance, centralized stress fibers indicate an increased centripetal force associated with Rho-A activation, whereas peripheral or cortical fibers reflect junctional preservation associated with Rac-1 activation [51–53]. The generation of centripetal tension is dependent on the activity of non-muscle myosin light chain (MLC) kinase, an enzyme required to phosphorylate MLC leading to the contraction of the actin-myosin fibers [54]. Rho-A regulates the activity of pMLC through a Rho-associated kinase (ROCK) that inhibits the activity of a MLC phosphatase preventing the relaxation of actin-myosin fibers [54, 55]. In support of this notion, we were able to block Rho-A-induced pMLC activation and the corresponding permeability changes with ROCK inhibitors. Of interest, Rho-A activation was associated with the loss of tight junctions in brain microvascular endothelial cells in patients with HIV-encephalitis [56]. Finally, as reported in other endothelial cell types [57], we found that Src plays a critical role modulating the permeability of cultured HGEc-1, since both Rho-A and Src inhibitors were needed to block the permeability changes.

It is worth discussing that the permeability changes described in cultured RGEc do not mimic the *in vivo* situation of the glomerular filtration barrier. The glomerular filtration barrier is comprised of fenestrated endothelial cells, the basement membrane, and podocytes with their foot processes and slit diaphragms [58–60]. Podocytes play a central role regulating the permeability of the glomerular filtration barrier, and previous studies showed that the cytoskeletal properties of HIV-podocytes are impaired [61]. However, the glycocalix that covers the fenestrations and endothelial cell bodies, and the HSPG located in the glomerular basement membrane, also offer resistance to the filtration of water and small molecules [58–60]. The role of the anionic sites of HSPG is documented by the rapid effacement of podocytes in rats kidneys perfused with protamine sulfate, and by the rapid reversal of these changes within minutes of perfusion with the polyanion heparin [62, 63]. Therefore, glomerular endothelial cells and podocytes may undergo similar cytoskeletal changes *in vivo*, and these changes could potentially affect the size and charge of the fenestrations, the thickness of the glycocalix, and the structure and function of podocytes. Further studies are needed to confirm this notion.

VEGF-A is secreted by podocytes and transported by diffusion across the glomerular basement membrane, where it plays a key role maintaining the integrity of glomerular endothelial cells [32]. HIV+ podocytes secrete high levels of VEGF-A in the urinary space [11]. In addition, FGF-2 released into the circulation of HIV+ children is accumulated in renal glomeruli bound to HSPG [13–15], and transported by convective flux to the urinary space. Therefore, as expected, we found high urinary levels of FGF-2 and VEGF-A in children with HIV-RD. Furthermore, previous studies showed that HIV-Tat and FGF-2, acting in a synergistic manner, increased the activity of Rho-A in podocytes cultured from the urine of children with HIVAN, and precipitated the development of HIV-nephropathy in HIV-Tg_26_ mice [30]. We should mention however, that podocytes cultured from children with HIV-RD may behave differently from normal podocytes, and that more studies are needed to determine how podocytes from HIV-negative children will respond to FGF-2 and VEGF-A. Nonetheless, studies in other transgenic mouse models showed that chronic activation of Rho-A in podocytes causes proteinuria [64, 65]. Alternatively, chronic Rac-1 activation or deletion specifically in podocytes, also causes proteinuria and glomerular disease [66, 67], and Rac-1 appears to be essential to maintain the normal structure of endothelial cells *in vivo* [68]. Taken together, all these findings suggest that changes in Rho-A and Rac-1 activity could play an important role in the pathogenesis of renal diseases associated with high circulating levels of FGF-2 and VEGF-A [11, 14, 69, 70].

In conclusion, we found that FGF-2, VEGF-A, and HIV-Tat, induced significant cytoskeletal changes and increased the permeability of cultured HGEc, acting through Rho-A, Rac-1, and Src. Moreover, since other factors released into the circulation and urine of HIV+ children, including TNF-α and thrombin, are also capable of inducing Rho-A activity in endothelial cells [71, 72], we speculate that the chronic and synergistic stimulation of these signaling pathways by multiple factors may accelerate the progression of HIV-RD in children. More studies are needed however, to define the clinical value of the REc assay described in this study to identify HIV+ children exposed to circulating factors that could induce glomerular injury through the induction of chronic changes in Rho-A, Rac-1 and Src activity.

## Supporting Information

S1 Fig The low levels of endotoxin lipopolysaccharide (LPS) detected in the urine samples of HIV-infected children do not affect the permeability of cultured HGEc. Urine samples harvested from HIV infected children with (HIV-RD) and without renal diseases (HIV-N) were used (1:10 dilution) to stimulate monolayers of cultured HGEc in presence or absence of LPS (25 pg/ml) and thrombin (100 units/ml) as a positive control. The data show changes in permeability assessed with FITC-dextran and expressed as fold increase in **(A)** primary human glomerular endothelial cells (HGEc), and **(B)** the glomerular endothelial cell line HGEc-1. Graph shows mean ± SEM corresponding to three different experiments (n = 5 samples per group). Values significantly different from the control samples were marked with *asterisks* **p<0.01. Values significantly different from HIV-N were marked with crosses + p<0.05 and ++p<0.01.
(DOCX)
