# Supplementary material for: Circulating Fibroblast Growth Factor-2, HIV-Tat, and Vascular Endothelial Cell Growth Factor-A in HIV-Infected Children with Renal Disease Activate Rho-A and Src in Cultured Renal Endothelial Cells
Source: PLoS One. 2016 Apr 20;11(4):e0153837. doi: 10.1371/journal.pone.0153837 (PMC4838216; doi:10.1371/journal.pone.0153837)
Supplement: S1 Fig — Urine samples harvested from HIV infected children with (HIV-RD) and without renal diseases (HIV-N) were used (1:10 dilution) to stimulate monolayers of cultured HGEc in presence or absence of LPS (25 pg/ml) and thrombin (100 units/ml) as a positive control. The data show changes in permeability assessed with FITC-dextran and expressed as fold increase in (A) primary human glomerular endothelial cells (HGEc), and (B) the glomerular endothelial cell line HGEc-1. Graph shows mean ± SEM corresponding to three different experiments (n = 5 samples per group). Values significantly different from the control samples were marked with asterisks **p<0.01. Values significantly different from HIV-N were marked with crosses + p<0.05 and ++p<0.01. (DOCX) [file pone.0153837.s001.docx]

**
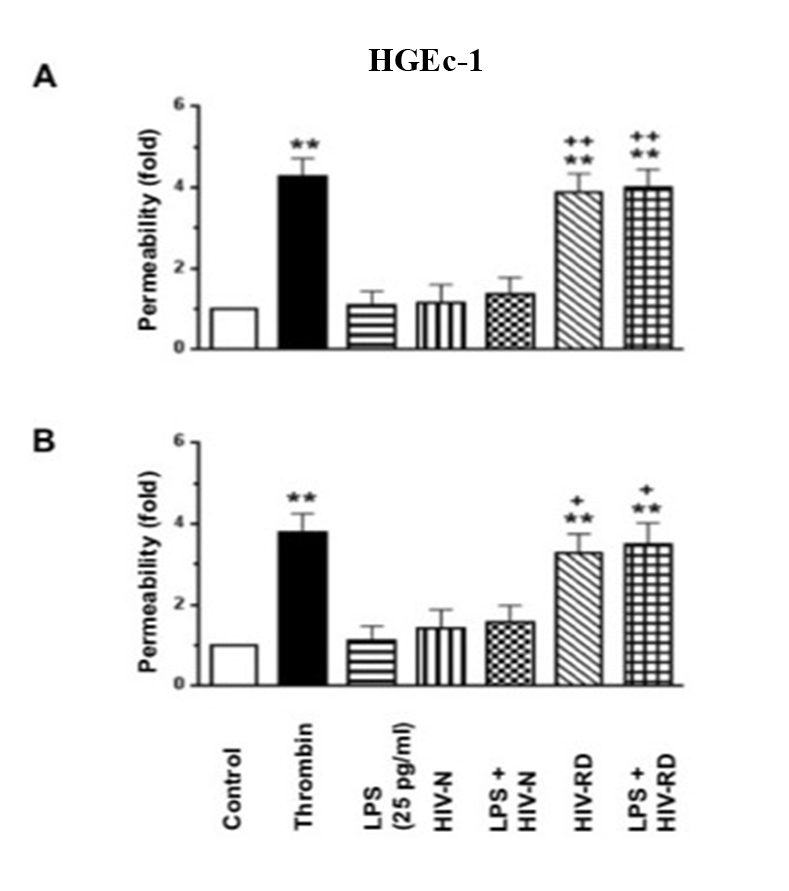
**

**S1 Fig. The low levels of endotoxin lipopolysaccharide (LPS) detected in the urine samples of HIV-infected children do not affect the permeability of cultured HGEc.** Urine samples harvested from HIV infected children with (HIV-RD) and without renal diseases (HIV-N) were used (1:10 dilution) to stimulate monolayers of cultured HGEc in presence or absence of LPS (25 pg/ml) and thrombin (100 units/ml) as a positive control. The data show changes in permeability assessed with FITC-dextran and expressed as fold increase in **(A)** primary human glomerular endothelial cells (HGEc), and **(B)** the glomerular endothelial cell line HGEc-1. Graph shows mean ± SEM corresponding to three different experiments (n= 5 samples per group). Values significantly different from the control samples were marked with *asterisks* **p<0.01. Values significantly different from HIV-N were marked with crosses + p<0.05 and ++p<0.01.
